# Identification of the circRNA-miRNA-mRNA Prognostic Regulatory Network in Lung Adenocarcinoma

**DOI:** 10.3390/genes13050885

**Published:** 2022-05-16

**Authors:** Yan Ma, Heng Zou

**Affiliations:** Shanghai Public Health Clinical Center, Fudan University, 2901 Caolang Road, Jinshan District, Shanghai 201508, China; 20211300002@fudan.edu.cn

**Keywords:** lung adenocarcinoma, circular RNA, ceRNA, prognosis, immune infiltration

## Abstract

Background: Numerous studies have identified that circular RNAs (circRNAs) can serve as competing endogenous RNAs (ceRNAs) to regulate tumor progression. However, there are still a large number of circRNAs to be deciphered. Objective: The purpose of this study was to reveal novel circRNAs and their potential role in lung adenocarcinoma (LUAD). Methods: To unveil LUAD-related circRNAs, microRNA (miRNAs), and messenger RNA (mRNA) and elucidate their possible molecular mechanisms, we employed a strategy combining extensive data mining and bioinformatics methods. According to the results of bioinformatics workflow analysis, a novel circRNA-miRNA-mRNA network was constructed. Results: Ten circRNAs with different expressions were acquired from four Gene Expression Omnibus (GEO) microarray datasets. Seven Prognostic-related differential miRNAs of LUAD were gained from The Cancer Genome Atlas (TCGA). Simultaneously, the miRNA reaction components corresponding to the ten circRNAs were predicted. Two circRNA–miRNA interactions including two circRNAs (hsa_circ_0008234 and hsa_circ_0002360) and two miRNAs (hsa-miR-490-3p and hsa-miR-1293) were identified above. Then, target genes of the two miRNAs and differently expressed genes (DEGs) from TCGA on LUAD were collected. Three hub-genes (*ADCY9*, *NMUR1*, *SYT1*) were determined according to prognosis in patients with LUAD ulteriorly. Conclusions: hsa_circ_0008234/hsa-miR-490-3p/*SYT1* and hsa_circ_0002360/hsa-miR-1293/ (*ADCY9*, *NMUR1*) networks were established, and identified molecules may be involved in pathogenesis and prognosis in patients with LUAD.

## 1. Introduction

Lung cancer is the first and most frequent cause of cancer-related death worldwide, partly due to its poor response to existing conventional target treatments or immune therapies [1]. Thus, the 5-year survival rate of patients and median overall survival (OS) remain poor depending on the different TNM stage of the tumor. Lung cancer is typically classified into about 85% non-small cell lung cancer (NSCLC) and about 15% small cell lung cancer (SCLC). In these subtypes, NSCLC were mainly refers to lung adenocarcinoma (LUAD) and squamous cell carcinoma (LUSC) [2]. Surgical excision is still the most effective treatment strategy for patients in the early stage. Combined with radiotherapy and chemotherapy, the postoperative survival rate of lung cancer patients can be significantly improved [3]. However, clinically successful treatment therapies are not very promising due to the lack of early diagnosis, postoperative tumor relapse, metastasis, and the development of drug resistance. Chemotherapy and targeted biological treatment were still the best for advanced lung cancer patients, especially LUAD patients. Therefore, exploring the genetic and epigenetic regulation of LUAD and finding new tumor molecular markers and therapeutic drugs play an important role in ameliorating the therapeutic effect and diminishing recurrence rate and mortality rate.

Circular RNAs (circRNAs) are a kind of endogenous non-coding RNA, which originates from the exon or intron region of a gene and forms a covalent closed-loop structure by reverse splicing. This structural characteristic makes circRNAs more stable and less degradable than linear RNAs [4]. Although a large number of previous studies have shown that circRNAs are vital in multiple cellular processions and human diseases, the complex biological functions, and possible molecular mechanisms are largely unexplored and elucidated. MicroRNAs (miRNAs) are also categorized as small ncRNAs, which can play a regulatory role after transcription of the target gene. In our previous studies, miRNAs such as Let-7a-5p could effectively inhibit the occurrence and development of NSCLC [5,6]. CircRNAs have harbored ample conserved miRNA response elements (MREs), which can act as competitive endogenous RNA (ceRNA) and bind to miRNA similar to a sponge, thus affecting the post-transcriptional regulation of miRNA on target genes. More importantly, numerous studies have indicated that abnormal expressions of circRNA play an indispensable role in the tumorigenesis, progress, invasion, and metastasis of lung cancer. For instance, circSATB2 promotes the expression of fascin actin-bundling protein 1 by sponging miR-326 to participate in the progression of NSCLC [7]. CircHIPK3 is highly expressed in lung cancer, and it could modulate the autophagy process by sponging miR-124-3p [8]. Additionally, Circular RNA cESRP1 [9], circular RNA CDR1 [10] and hsa_circ_00001905 have also been proved to be candidate biomarkers and molecular targets for clinical therapy in NSCLC [11]. Research into circRNAs is becoming a promising hotspot in the RNA field due to its multiple functions and specific characteristics. Nevertheless, many circRNAs remain to be deciphered in LUAD. Consequently, for the sake of further understanding and illuminating the potential role of circRNAs in LUAD, we identified differentially expressed circRNAs (DECs) through bioinformatics methods and then constructed a regulatory circRNA-miRNA-mRNA network.

The present study employed a combinative strategy of extensive data mining and bioinformatic methods to clarify LUAD-related circRNAs, microRNA (miRNAs), and messenger RNA (mRNA) and to elucidate their possible mechanisms. First, LUAD-related circRNA microarray datasets were obtained from the GEO database, and the GEO2R online analysis tool was used to acquire DECs. In addition, based on differentially expressed miRNAs (DEMs) expression profiling from the TCGA database, a prognostic model was built to obtain related miRNAs by using the least absolute shrinkage and selection operator (LASSO) algorithm and Cox regression. To ascertain whether the DECs function as ceRNAs in LUAD, the corresponding circRNA-miRNA interactions were constructed by combining with miRNAs predicted by the website and miRNAs related to prognosis, and miRNA target genes were collected to build a circRNA-miRNA-mRNA network. Subsequently, Gene Oncology (GO) functional enrichment, Kyoto Encyclopedia of Genes and Genomes (KEGG) pathway enrichment analyses on the target genes were executed to expound on the underlying pathogenesis of LUAD. Simultaneously, the protein–protein interaction (PPI) network was successfully established, and the hub genes were also determined. Finally, the tumor infiltration by immune cells was deemed to be a reasonable interpretation for the relationship between hub gene expression and survival.

## 2. Methods

### 2.1. Source of LUAD Datasets

The microarray datasets providing circRNA expression profiles for human samples in lung cancer were obtained from the GEO database. We selected GSE101684, GSE 101586, GSE112214, and GSE158695. The GSE101684 dataset contains four LUAD tissues derived from the inferior lobe of the right lung and four para-cancerous tissues. The GSE 101586 has five LUAD tissues and five normal lung tissues. The GSE 112214 and GSE 158695 include three NSCLC samples and three normal lung tissues. The details of four circRNA microarray data are shown in Table 1. MiRNA and mRNA expression profile data, and clinical information were obtained from TCGA, including 521 LUAD tissue samples and 46 adjacent normal tissues. LUAD tissue with missing clinical information were excluded, and 500 LUAD samples were shortlisted for further analysis.

### 2.2. Differential circRNA Expression and Venn Analysis

We used GEO2R online analysis tool on the NCBI website and Venn diagram software to obtain the mutual DECs in the four GEO datasets above. The criteria for the significant differentially expressed were |log2(FC)| > 1 and adjusted *p* < 0.05. In this study, we selected circRNAs expression profiles that were co-highly expressed in three or four GEO databases by Venn analysis. Meanwhile, the commonly significant lower expression circRNAs in the three GEO datasets were obtained and the simultaneously lower expressed were screened out through the Venn analysis.

### 2.3. Definition of the miRNAs-Related Prognostic Model

To verify the association between the survival time and target miRNAs from the TCGA database, we carried out the Univariate Cox, LASSO, and multivariate Cox regression analyses via the glmnet (version 2.0.18) and survival (version 2.44.1.1) R packages. First, univariate Cox regression was performed to determine the correlation between miRNAs expression levels and overall survival. A *p* value less than 0.05 is considered a criterion, which indicates that the difference was statistically significant. Then, the LASSO regression analysis was performed on these miRNAs that met this criterion. In this way, we can further screen for candidate prognostics miRNAs, the minimum λ value was used as the inclusion criteria and can represent this model’s most appropriate number of variables. The miRNAs screened by the above methods were further analyzed by multivariate Cox regression to evaluate the independent contribution of each miRNA to prognosis and hazard ratios (HR), and 95% confidence intervals (CI) for the key miRNAs were calculated. According to the formula: Σ (expmiRNAn × βmiRNAn), we used the Cox regression coefficient (β) and expression levels of miRNAs to calculate prognosis-related risk scores. The LUAD samples were divided into high-risk and low-risk groups based on risk score.

### 2.4. Evaluation of the Risk Model and Identification of Potential Biomarkers

Time-dependent receiver operator characteristic (ROC) curves were established, and the area under the curve (AUC) was calculated to assess the performance of the prognostic model above. DEMs survival times were plotted by K m survival analyses. Multivariate Cox regression were performed to ensure which miRNAs differentially expressed could serve as potential prognostic molecular markers. The above indicators with statistically significant differences were *p* values < 0.05.

### 2.5. Prediction of miRNA Binding Sites and Target mRNAs

The potential target miRNA of the circRNAs were predicted using the circbank (http://www.circbank.cn/searchMiRNA.html accessed on 1 May 2021). At the same time, we selected the DEMs through the above prognosis model analysis. Next, we conducted a Venn diagram analysis; overlapping predicted miRNAs were chosen for further investigation and research. Furthermore, we used miRwalk (http://mirwalk.umm.uni-heidelberg.de/ accessed on 1 May 2021) to predicate target mRNAs of these selected miRNAs, which can bind to the target mRNA 3′-UTR. The differentially expressed genes (DEGs) in LUAD were gained from the TCGA database. Similarly, overlapping predicated mRNAs were selected for the following bioinformatics analysis.

### 2.6. GO and KEGG Pathway Analyses

We used DAVID (https://david.ncifcrf.gov/ accessed on 1 May 2021) bioinformatics resources to carry out GO, and KEGG pathway analysis of the overlapping predicted differential expressed mRNAs. The criteria for the statistically significant differential expressed is a *p* value < 0.05.

### 2.7. Establishment of PPI Network and Identification of Hub-Genes

We constructed a PPI network for targeted DEGs via using the STRING (https://cn.string-db.org/ accessed on 1 May 2021) online website. Then, PPI network was embellished through Cytoscape software. The Cytoscape plug-in cytoHubba was used to screen hub genes with the node degree. The Cytoscape plug-in MCODE to screen out the essential modules in the PPI network with the selected criteria as follows: degree cut-off = 2, node score cut-off = 0.2, Max depth = 100, and k-score = 2.

### 2.8. Differentially Expressed and OS Analysis of the Hub Genes

The differential expression data of these hub genes in LUAD tissues and normal tissues were gained using the TCGA. The effect of these hub genes on OS rate in lung cancer was identified using the Gene Expression Profiling Interactive Analysis (GEPIA, http://gepia2.cancer-pku.cn/#index accessed on 3 May 2021), a web-based tool derived from the TCGA.

### 2.9. Correlation Analysis of Hub Gene and Identification of the Association between Hub Gene and Immune Infiltration in LUAD

Based on the GEPIA, we determined the correlation of the hub genes mentioned above with each other. Meanwhile, the Tumor Immune Estimation Resource (TIMER, http://timer.cistrome.org/ accessed on 3 May 2021) website was used to analyze the correlation between the expression of hub genes and the infiltration of several immune cells in LUAD.

## 3. Results

### 3.1. Differentially Expressed circRNAs Analysis in the NSCLC

**Figure 1:**
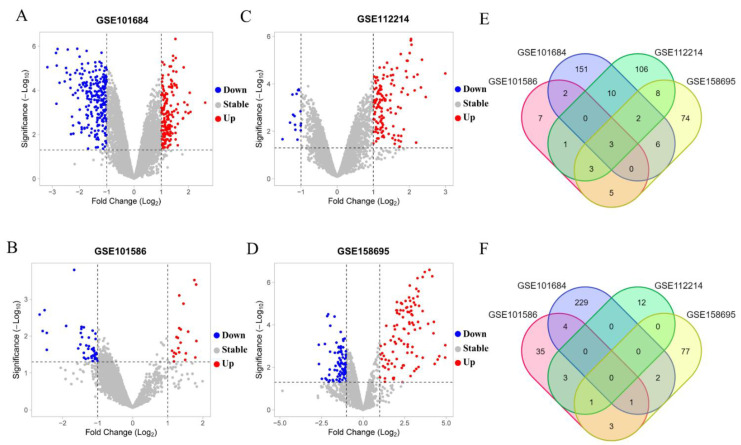
Volcano plots for of differentially expressed circRNAs in LUAD, the red points and blue points represent up and down expressed circRNAs, respectively. (**A**) GSE101684, (**B**) GSE101586, (**C**) GSE112214, (**D**) GSE15869. The eight upregulated circRNAs (**E**) and two downregulated (**F**) circRNAs were identified by intersection of circRNAs from the four databases.

With a *p* value < 0.05, |log2(FC)| > 2 as the standard of statistical difference, we selected a total of 813 DECs from four microarray datasets (GSE101684, GSE101586, GSE112214, GSE158695). The principal information for the four datasets is presented in Table 1. A total of 411 DECs were identified in the GSE101684 dataset, 175 circRNAs were upregulated and 236 circRNAs were downregulated (Figure 1A); 68 DECs with 21 were upregulated and 47 were downregulated and identified from the GSE101586 dataset (Figure 1B); 149 DECs were determined in the GSE112214 dataset, 133 of which were upregulated and 16 were downregulated (Figure 1C); 101 upregulated circRNAs and 84 downregulated circRNAs were found derived from the GSE158695 dataset (Figure 1D). Then, we used the Venn diagram analysis tool to identify the overlapped circRNAs from the four microarray datasets. The upregulated circRNAs shared in three or four data sets were selected for further analysis (hsa_circ_103415, hsa_circ_101066, hsa_circ_104513, hsa_circ_103134, hsat_circ_100395, hsa_circ_101213, hsa_circ_102046, hsa_circ_102442) (Figure 1E). Similarly, circRNAs downregulated in all three data sets were selected for further analysis (has_circ_002178, has_circ_103123) (Figure 1F). The details concerning the selected circRNAs were listed in Table 2.

### 3.2. Prognostic-Related Differential miRNAs from TCGA

**Figure 2:**
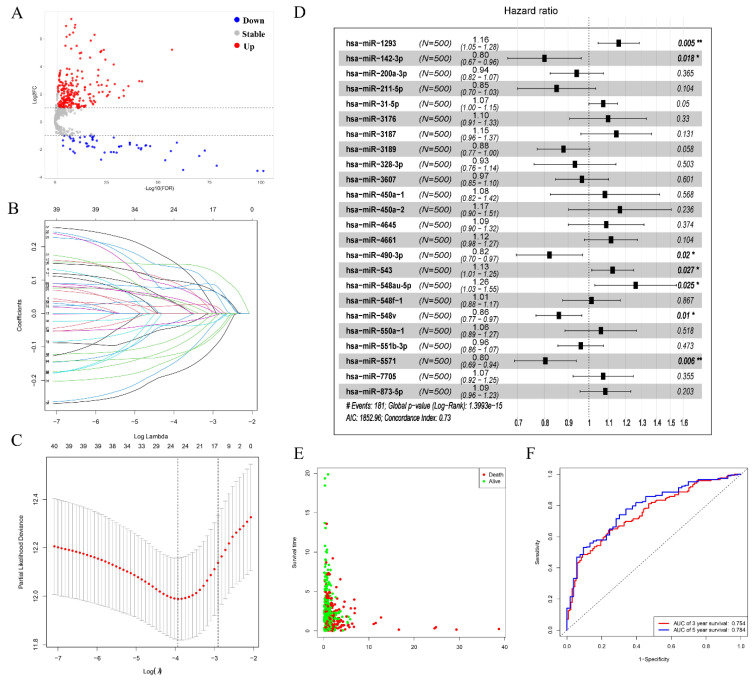
Prognostic-related differential miRNAs from TCGA. (**A**) Volcano plots for DEMs in LUAD derived from the TCGA database. the red points and blue points represent up and down expressed miRNAs, respectively. (**B**,**C**) Determination of the number of factors by the LASSO analysis, LASSO coefficient profiles of 41 prognostic miRNAs. (**D**) Forest plot displaying the hazard ratio and 95% confidence interval for the key prognostic miRNAs. (**E**) Survival status of LUAD patients. (**F**) Time-dependent receiver operator characteristic curves for OS prediction at 3- and 5-years. “*” means *p* < 0.05, “**” means *p* < 0.01.

Based on the miRNA expression profile obtained from the TCGA database and the cut-off criterion of |log2(FC)| > 2, *p* < 0.05, we screened out 298 significantly DEMs in the LUAD patient sample (Figure 2A). Among these selected miRNAs, including 241 upregulated and 57 downregulated miRNAs, these miRNAs were selected for predicting prognosis analysis. Firstly, we used the univariate Cox regression to evaluate the correlation between DEMs and patients’ OS. 41 miRNAs have an obvious correlation with OS of patients. Then, LASSO regression analysis was performed on these miRNAs. Ultimately, 25 miRNAs were determined for further analysis when the λ value was at a minimum (Figure 2B,C). At the same time, we performed a multivariate Cox regression analysis to acquire HR values and 95% CIs for selected miRNA; the results showed that seven prognostic-related differential miRNAs of LUAD were obtained (hsa-miR-1293, hsa-miR-142-3p, hsa-miR-490-3p, hsa-miR-543, hsa-miR-548au-5p, hsa-miR-548v, hsa-miR-5571) (Figure 2D). All specimens were divided into two groups: high-risk group and low-risk group (Figure 2E). We drew a time-dependent ROC curve according to the above median risk score values. The AUCs for 3- and 5-year survival were 0.754 and 0.784, respectively, demonstrating that the risk score model has a stable performance (Figure 2F).

### 3.3. Prediction of Target miRNAs and Identification of circRNA-miRNA Interactions

**Figure 3:**
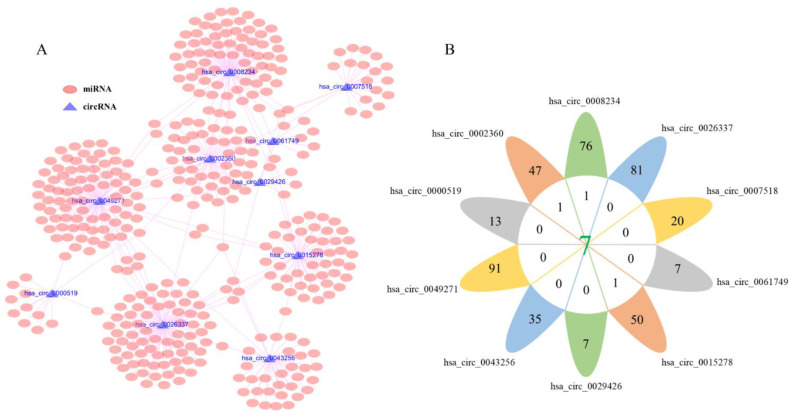
Identification of circRNA–miRNA interactions. (**A**) Prediction of target miRNAs of DECs via using the circbank, the pink oval represents miRNAs, blue triangle represents circRNA. (**B**) Venn diagram analysis and 3 miRNAs were determined.

**Figure 4:**
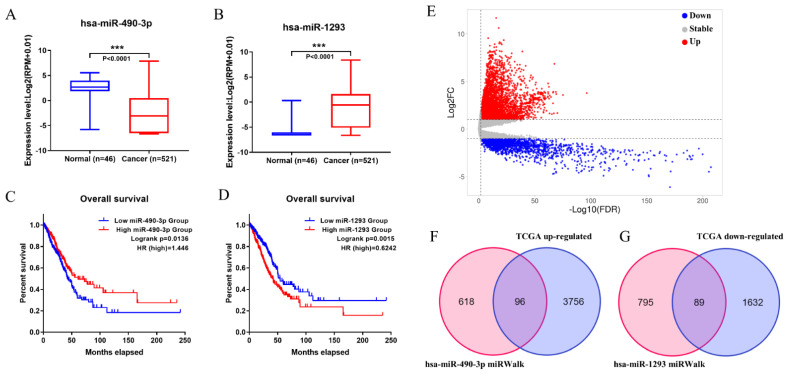
The expression level and OS time of 2 miRNAs, and target genes were predicted. (**A**,**C**) has-miR-490-3p. (**B**,**D**) has-miR-1293. (**E**) Volcano plots for differentially expressed mRNAs in LUAD derived from the TCGA database. (**F**,**G**) Intersecting the predicted target genes and DEGs, respectively. “***” means *p* < 0.001.

All the possible target miRNAs above the selected dec were identified through the online website of circbank: hsa_circ_0008234 (77 target miRNAs), hsa_circ_0026337 (81 target miRNAs), hsa_circ_0007518 (20 target miRNAs), hsa_circ_0061749 (7 target miRNAs), hsa_circ_0015278 (51 target miRNAs), hsa_circ_0029426 (7 target miRNAs), hsa_circ_0043256 (35 target miRNAs), hsa_circ_0049271 (91 target miRNAs), hsa_circ_0000519 (13 target miRNAs), and hsa_circ_0002360 (48 target miRNAs) are shown in (Figure 3A). The above-mentioned miRNAs related to prognosis obtained from TCGA were analyzed by Venn diagram, and three miRNAs (hsa-miR-142-3p, hsa-miR-490-3p, hsa-miR-1293) were determined (Figure 3B). In addition, considering that the expression level of hsa-miR-142-3p did not conform to the mechanism of ceRNA, it was excluded. The expression levels of the hsa-miR-490-3p and hsa-miR-1293 in LUAD are exhibited (Figure 4A,B), and K–M survival curves were performed to describe their effects on the OS (Figure 4C,D). The results demonstrated that hsa-miR-490-3p was the shallow expression in LUAD and had a better prognosis when overexpressed. It may be a tumor suppressor miRNA. On the contrary, hsa-miR-1293 may be a novel oncogenic miRNA in LUAD. In conclusion, hsa-miR-490-3p and hsa-miR-1293 have potential prognostic biomarkers for LUAD patients.

### 3.4. Construction of circRNA-miRNA-mRNA Network

**Figure 5:**
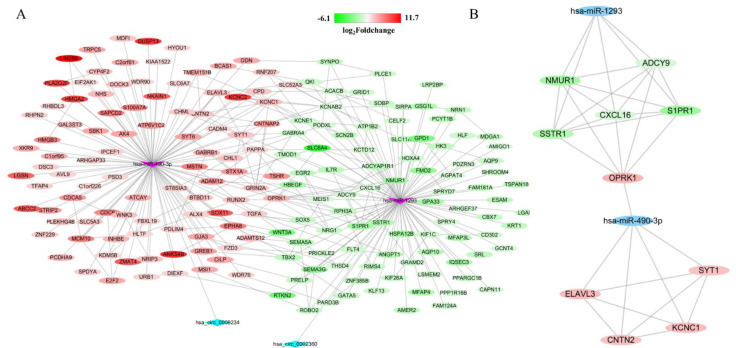
Construction of circRNA–miRNA–mRNA regulatory network and PPI network, and identification of hub-genes from network with the MCODE algorithm. Gradual changes in color according to the log2(foldchange) of genes in LUAD. (**A**) The network consisting of 2 cricRNAs, 2 miRNAs and 185 genes was generated by Cytoscape. (**B**) A network of 10 hub-genes and 2 miRNAs.

Firstly, a total of 5388 DEGs were obtained from the TCGA database, including 3852 upregulated and 1721 downregulated genes (Figure 4E). Additionally, 714 target mRNAs of hsa-miR-490-3p and 884 target mRNAs of hsa-miR-1293 were obtained from the miRWalk. We identified 96 upregulated and 89 downregulated target genes that play an essential role in LUAD by cross-predicting the target gene and DEGs, respectively (Figure 4F,G). Subsequently, the circRNA-miRNA interplay, and miRNA–mRNA interplay were integrated to establish a circRNA–miRNA–mRNA network (Figure 5A), which primarily shed light on the correlation between the DECs (hsa_circ_0008234 and hsa_circ_0002360), miRNAs (hsa-miR-490-3p and hsa-miR-1293) and the 185 mRNAs. The PPI network was constructed using STRING and Cytoscape to gain insight into the biological interactions of 185 genes and to define the circRNA-miRNA-mRNA regulatory network (Figure 5A). Then, we used the plug-in of CytoHubba in Cytoscape to screen out the top 10 node degrees to represent the central genes of the PPI network, including *NMUR*, *ADCY9*, *CXCL16*, *SSTR1*, *S1PR1*, *OPRK1*, *ELAVL3*, *SYT1*, *CNTN2* and *KCNC1*(Figure 5B).

### 3.5. Functional Enrichment Analysis

**Figure 6:**
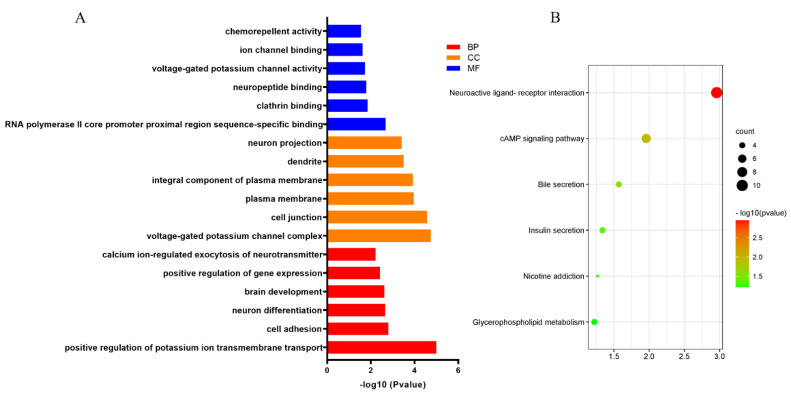
Functional enrichment analysis. (**A**) Top six significant GO enrichment annotations of the 185 overlapped genes, including BP, CC, MC. (**B**) Top six significant KEGG pathways. The degree of enrichment of displayed through color gradual changes of the node. The size of the dots represents gene counts in a pathway.

GO and KEGG analyses were performed by DAVID online database to evaluate the function of the target mRNAs of these two DEMs in the subnetwork. The top six enriched terms under the three common categories: biological process (BP), molecular function (MF), and cellular component (CC), were shown (Figure 6A). Results from the first six BP-enriched terms revealed that the target mRNAs were primarily associated with “calcium icon-regulated exocytosis of neurotransmitters”, “positive regulation of gene expression”, “brain development”, “neuron differentiation”, “cell adhesion” and “positive regulation of potassium ion transmembrane transport”. The top six KEGG pathways showed the target mRNAs most likely to be involved in the “cGMP-PKG singling pathway”, which provides more evidence in support of this assumption that DECs may play vital roles in cancer initiation and progression, and metastasis.

### 3.6. Identification of Hub Genes

**Figure 7:**
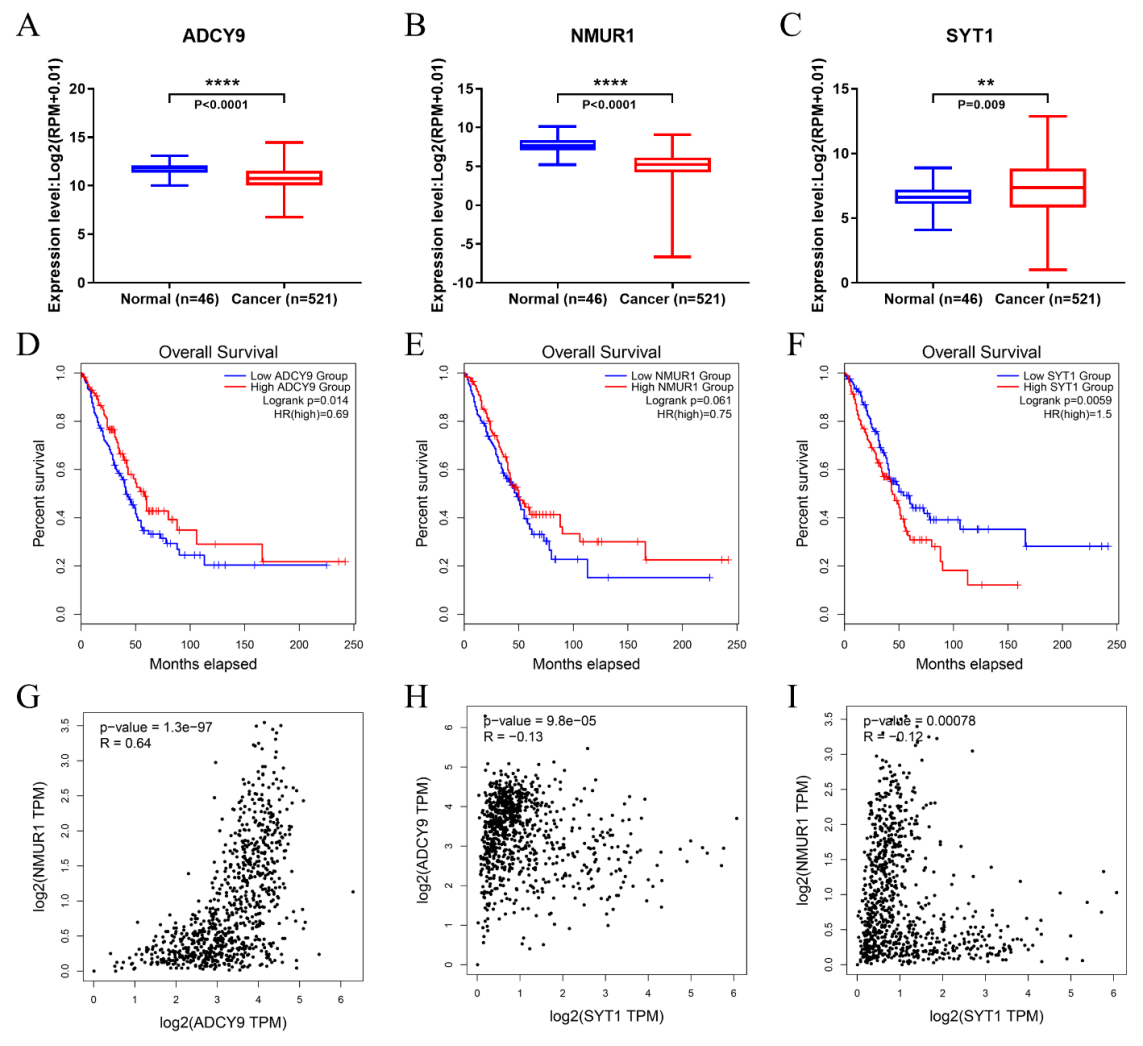
The expression level and OS time of 3 hub-genes, and correlation analysis for three differentially expressed hub-genes. (**A**,**D**) *ADCY9*. (**B**,**E**) *NMUR1*. (**C**,**F**) *SYT1*. (**G**) The correlation of *ADCY9* with *NMUR1* in LUAD. (**H**) The correlation of *ADCY9* with *SYT1* in LUAD. (**I**) The correlation of *NMUR1* with *SYT1* in LUAD. “**” means *p* < 0.01, “****” means *p* < 0.0001.

The relative expression level of the top ten hub genes and the effect on OS were obtained from the TCGA. Only three hub genes (*ADCY9*, *NMUR1*, *SYT1*) are related to the overall survival of patients. Specifically, LUAD patients with overexpressed *SYT1* (Figure 7A,D) have a low OS rate, which indicates it can be a candidate for poor prognostics. Furthermore, the downregulated hub genes *ADCY9* (Figure 7B,E) and *NMUR1* (Figure 7C,F) in the LUAD negatively affected the OS rate, which may be a favorable prognostics factor. Moreover, using the GEPIA, we examined the correlation of three differentially expressed hub genes and found that the expressions of *ADCY9*, *NMUR1* and *SYT1* were strongly associated with each other in LUAD (Figure 7G–I).

### 3.7. The Hub Genes Expression Was Correlated yo Immune Cell Infiltration in LUAD

**Figure 8:**
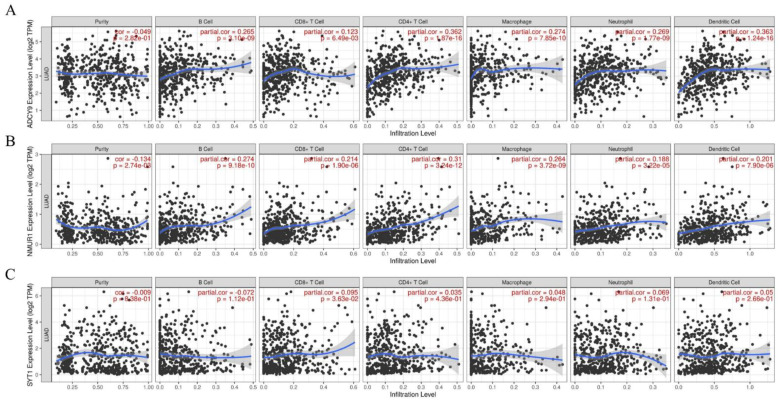
Correlation analysis of 3 hub-genes expression and tumor-infiltrating immune cells. (**A**) *ADCY9*. (**B**) *NMUR1*. (**C**) *SYT1*.

The analysis of the TIMER website indicated that the expression of three hub genes (*ADCY9*, *NMUR1*, *SYT1*) was significantly associated with the infiltration of several immune cells in LUAD, including B cells, CD4+ T cells, CD8+ T cells, neutrophils, macrophages, and DCs. The result expounded that the expression of *ADCY9* (Figure 8A) and *NMUR1* (Figure 8B) were positively correlated with B cells, CD4+ T cells, CD8+ T cells, neutrophils, macrophages, and DCs. Thus, we speculated that the high expression of *ADCY9* and *NMUR1* might facilitate the anti-tumor immunity in the LUAD microenvironment. This result also proved that patients with high expression of *ADCY* and *NMUR1* genes had a high survival rate. On the contrary, we found no significant correlation between the *SYT1* (Figure 8C) gene expression and immune cells infiltration. Therefore, *SYTI* may be a proto-oncogene that may inhibit the anti-tumor immune response and is negatively correlated with patient survival time.

## 4. Discussion

Human genes can be divided into protein-coding genes and non-coding genes. Although more than 90% of human genes can be actively transcribed, the known protein-coding genes only account for about 1.1% of the human genome, while the majority of the gene transcription variants may be classified as ncRNAs [12,13]. In the past few years, many ncRNAs have been identified in human tissues, body fluids and cells to benefit from the development of high-throughput sequencing and genomic analysis platforms. Numerous studies have suggested that these ncRNAs have various biological functions and possibly participate in multiple metabolic pathways [14]. Compared with known ncRNAs, circRNA is a novel research hotspot and has gained popularity among researchers. In addition, due to the biological properties of circRNAs being covalently closed loops, circRNAs can be more stable existing in tissue, cells, or plasma than other ncRNAs [15]. It has been reported that circRNAs play various essential roles in biological functions, including microRNA sponges, RBP-binding molecules, transcriptional regulation factors, or protein translation templates [16,17]. Recent studies have indicated that a large number of circRNAs are differentially expressed in specific tumor cells and tissues, which may be involved in occurrence and development of tumors. In these studies, one of the biological functions of circRNA that has been studied extensively is that it can act as a sponge of miRNA to regulate gene expression to promote or inhibit the occurrence and progression of tumors [18]. For example, the upregulated circRNA circSEPT9 in TNBC could release the inhibition of leukocyte inhibitor factors by downregulating mir-637 and accelerate the carcinogenesis, development and metastasis of triple-negative breast cancer [19]. Furthermore, some circRNAs may serve as a tumor suppressor to inhibit tumor growth, invasion, and metastasis, so the downregulation of circRNAs may exert opposite effects. For example, circ-HuR was determined to be a tumor suppressor circRNA, can suppress HuR expression and gastric cancer progression, and can be a candidate for a potential therapeutic target for gastric cancer [20]. Circular RNA hsa_circ_0000326, a novel circRNA found in lung cancer patient microarray, can act as a miR-338-3p sponge and alter the function of miR-338-3p to facilitate LUAD proliferation and metastasis [21].

In this study, we investigated the circRNA expression profile in LUAD tissues and para-cancerous tissues from four GEO databases (GSE101684, GSE112214, GSE 101586, and GSE158695). We focused on differentially expressed circRNAs in lung cancer tissues, which were remarkably upregulated or downregulated in cancer tissues, and may be significantly related to tumor occurrence, development, invasion, and metastasis. Here, we selected eight upregulated circRNAs (hsa_circRNA_103415, hsa_circRNA_101066, hsa_circRNA_104513, hsa_circRNA_103134, hsa_circRNA_100395, hsa_circRNA_101213, hsa_circRNA_102046, and hsa_circRNA_102442) and two downregulated circRNAs (hsa_circRNA_002178 and hsa_circRNA_103123) for further analysis. As highly conserved covalently closed RNAs, these selected circRNAs may contain abundant miRNA binding sites, indicating that they can serve as a sponge to adsorb the corresponding miRNA and thus function as ceRNAs to regulate correlated protein gene expression. Herein, we found the possible miRNAs binding sites of these ten dysregulated expressed circRNAs by circBank. At the same time, according to the data derived from the TCGA database, survival analysis and prognosis assessment of DEMs in LUAD were performed to construct a prognosis model and identify a potential miRNA biomarker. Finally, we determined hsa-mir-490-3p and hsa-mir-1293 in overlapped miRNAs for further analysis. Our prognosis model proved that the upregulated mir-490-3p could be associated with a good prognosis in LUAD. Previous studies have revealed that mir-490-3p can serve as tumor suppressor miRNA, and it is related to the occurrence and progression of various tumors. Zhiyong et al. demonstrated that miR-490-3p inhibited the Malignant Progression of LUAD cells by downregulating the Wnt/β-catenin signaling pathway [22]. Kang et al. reported that mir-490-3p was downregulated and mir-490-3p can promote cell proliferation, metastasis, and invasion via targeting HMGA2 [23]. In contrast, mir-1293 is considered a marker of poor prognosis and adverse to patient survival time. Although, Takagawa et al. observed that mir-1293 can suppress tumor progression by inhibiting DNA repair pathways and suggested that miR-1293 is a candidate for developing miRNA-based cancer therapeutics [24]. The molecular mechanism of miR-1293 in LUAD has not been reported yet. Next, the target genes of mir-490-3p and mir-1293 were identified through the miRwalk and TCGA database. Functionally, GO and KEGG pathway analysis was conducted for the target mRNAs to further investigate the underlying biological pathway of these target genes. A total of 185 target mRNAs were enriched in BP, MF, and CC terms, including “positive regulation of gene expression”, “cell junction”, “cell adhesion”, and “RNA polymerase II core promoter proximal region sequence-specific binding”, The results of KEGG pathway analysis indicated that “cGMP-PKG signaling pathway” was significantly enriched. Subsequently, we constructed a PPI network, screening ten hub genes from the PPI network. We investigated the expression of these genes in lung cancer tissues from the TCGA database and analyzed the association between both hub genes and patient survival time. The results showed that the downregulation of *ADCY9* and *NMUR1* in LUAD can inhibit the growth and aggressiveness of cancer, while the upregulation of *SYT1* had the contrary effects. Additionally, the expressions of *ADCY9* and *NMUR1* in LUAD showed a positive correlation, while the expressions of *SYT1* and *ADCY9*, *SYT1* and *NMUR1* showed a negative correlation. As for the functions of the selected hub genes, mutations in *SYT1*, the master switch responsible for allowing the human brain to release neurotransmitters, could lead to a rare neurodevelopmental disorder [25]. Recent studies have shown that SYT1 is also involved in cancer regulation [26,27]. ADCY9 is a widely distributed adenylate cyclase that catalyzes formation of cyclic AMP from ATP [28]. In addition, studies have demonstrated that ADCY9 takes to participate in the regulation of cellular function in certain cancers and drug responses [29,30]. NMUR1 is widely distributed in various organs of the human body and participates in the regulation of activation of phospholipase C activity, chloride transport, second messenger-mediated signaling and other physiological functions, and also plays an important role in the occurrence and development of various cancers [31]. However, there is little research on these hub genes in LUAD. Further experiments are needed to prove that hub genes participate in tumorigenesis. The microenvironment of a tumor consists of heterogeneous populations, including cancer cells themselves, infiltrating immune cells, and stromal cells; tumor-infiltrating immune cells play a vital role in tumorigenesis, progression, and metastasis, which not only inhibit tumor progression by attacking and killing cancer cells but also promote tumor progression by changing the tumor microenvironment [32,33]. With the development and application of new technologies such as single-cell RNA sequencing and mass cytometry, emerging evidence has shown that immune cells within the tumor microenvironment may possess various functions besides conventional tumor-antagonizing functions [34,35]. Hence, we assess the association between three hub genes and immune cells, respectively. The results demonstrated the positive correlation between the expression of *ADCY9* and *NMUR1* and immune cells. Therefore, the dysregulated expression and functional impairment of *ADCY* and *NMUR1* could affect the anti-tumor immune reaction of the body’s immune system. However, the *SYT1* gene showed the opposite result to the two genes mentioned above. Based on the above analysis results, we speculated that these hub genes might become new targets for immunotherapy or potential tumor immunomarkers.

## 5. Conclusions

In this study, a regulatory network of circRNA-miRNA-mRNA was successfully constructed. Our work demonstrated that some novel circRNAs, miRNAs, and hub genes may have clinical application value as prognostic markers or novel biomarkers in LUAD. Although the current research has certain limitations, the combination of molecular signaling pathway mechanism research and bioinformatics analysis to identify potential cancer diagnostic biomarkers and therapeutic targets still has extensive advantages. Thus, the above evidence indicated that regulatory network analysis of circRNAs could be a powerful tool to explore the molecular mechanism of circRANAs, miRNAs, and hub genes in LUAD development. Additionally, we found that the hub gene may affect the immune response of the body, and it can become a candidate gene for immunotherapy targets or potential tumor immune markers. Nevertheless, further validation of the biological function and mechanism of these circRNAs, miRNAs, and hub genes need to be carried out to assess whether they can serve as novel biomarkers or therapeutic targets in LUAD patients to improve the diagnosis and treatment of LUAD.

## Figures and Tables

**Table 1:** Essential characteristics of the four GEO datasets.

Data Source	Platform	First Author	Year	Region	Sample Size (T/N)	Number of circRNAs	Title
GSE101684	GPL21825	Ming Xu	2019	China	4/4	9114	CircRNA expression profiles in early stages lung adenocarcinoma.
GSE112214	GPL19978	Yeping Dong	2019	China	3/3	4407	Genome-wide analysis of circRNA expression profile in non-small cell lung cancer (NSCLC) tissues and matched adjacent normal tissues.
GSE158695	GPL19978	Botai Li	2020	China	3/3	1941	circRNA expression data from human non-small cell lung cancer tissues and the correponding non-cancerous tissues.
GSE101586	GPL19978	Mantang Qiu	2017	China	5/5	4425	Profiling circular RNA expression in lung cancer.

**Table 2:** Differentially expressed circRNAs that overlapped in all above-described GEO datasets.

CircRNA	Alias	Position	Best Transcript	Gene Symbol	Length	Regulation
hsa_circRNA_103415	hsa_circ_0008234	chr3: 71090478-71102924 strand: −	NM_001244808	FOXP1	587	Up
hsa_circRNA_101066	hsa_circ_0026337	chr12: 52180325-52188425 strand: +	NM_014191	SCN8A	853	Up
hsa_circRNA_104513	hsa_circ_0007518	chr7: 140402665-140404763 strand: +	NM_004546	NDUFB2	249	Up
hsa_circRNA_103134	hsa_circ_0061749	chr21: 40648098-40649277 strand: −	NM_033656	BRWD1	142	Up
hsa_circRNA_100395	hsa_circ_0015278	chr1: 173726114-173744981 strand: +	NM_014458	KLHL20	671	Up
hsa_circRNA_101213	hsa_circ_0029426	chr12: 131357380-131357465 strand: +	NM_006325	RAN	85	Up
hsa_circRNA_102046	hsa_circ_0043256	chr17: 35604934-35609962 strand: −	NM_198839	ACACA	483	Up
hsa_circRNA_102442	hsa_circ_0049271	chr19: 10610070-10610756 strand: −	NM_203500	KEAP1	686	Up
hsa_circRNA_002178	hsa_circ_0000519	chr14: 20811436-20811534 strand: −	NR_002312	RPPH1	98	Down
hsa_circRNA_103123	hsa_circ_0002360	chr21: 36206706-36231875 strand: −	NM_001001890	RUNX1	297	Down

## Data Availability

Data available in a publicly accessible repository. The data sets analyzed during the current study are available in the Gene Expression Omnibus (GEO, https://www.ncbi.nlm.nih.gov/geo/ accessed on 1 May 2021) and The Cancer Genome Atlas (TCGA, https://cancergenome.nih.gov/ accessed on 1 May 2021).
